# Perceptions and Practices of Community Pharmacists towards Antimicrobial Stewardship in the State of Selangor, Malaysia

**DOI:** 10.1371/journal.pone.0149623

**Published:** 2016-02-22

**Authors:** Muhammad Umair Khan, Mohamed Azmi Ahmad Hassali, Akram Ahmad, Ramadan Mohamed Elkalmi, Syed Tabish Razi Zaidi, Sameer Dhingra

**Affiliations:** 1 Department of Clinical Pharmacy, Faculty of Pharmaceutical Sciences, UCSI University, Kuala Lumpur, Malaysia; 2 Student Affairs & Networking, Discipline of Social and Administrative Pharmacy, School of Pharmaceutical Sciences, Universiti Sains Malaysia, Penang, Malaysia; 3 Department of Pharmacy Practice, Kulliyyah of Pharmacy, International Islamic University Malaysia, Kuantan Campus, Pahang, Malaysia; 4 Pharmacy, School of Medicine, Faculty of Health, University of Tasmania, Hobart, Tasmania, Australia; 5 School of Pharmacy, Faculty of Medical Sciences, The University of the West Indies, St. Augustine, Eric Williams Medical Sciences Complex, Uriah Butler Highway, Champ Fleurs, Trinidad and Tobago; University Medical Center Groningen, NETHERLANDS

## Abstract

**Background:**

Increasing antimicrobial resistance is one of the pressing concerns globally. Injudicious use of antibiotics is one of the modifiable factors responsible for antimicrobial resistance. Given the widespread use of antimicrobials in community settings, pharmacists have an important role in ensuring appropriate use of antibiotics. The objective of this study was to assess the perception and self-reported practices of community pharmacists towards antimicrobial stewardship.

**Methods:**

A cross-sectional study was conducted among community pharmacists between March–April, 2015, using a self-administered, pre-tested questionnaire in the State of Selangor, Malaysia. A simple random sampling approach was used to select pharmacy sites. Descriptive and inferential statistical methods were used to analyse the data.

**Results:**

A total of 188 pharmacists responded to the survey, giving a response rate of 83.5%. The majority of participants (n = 182, 96.8%) believed that antimicrobial stewardship program helps healthcare professionals to improve the quality of patient care. However, more than half of pharmacists were neutral in their opinion about the incorporation of antimicrobial stewardship programs in community pharmacies (n = 102, 54.2%). Though collaboration was often done by pharmacists with other health professionals over the use of antibiotics (n = 104, 55.3%), a significant proportion of participants (n = 102, 54.2%) rarely/occasionally participate in antimicrobial awareness campaigns. Pharmacists having postgraduate qualification were more likely to held positive perceptions of, and were engaged in, antimicrobial stewardship than their non-postgraduate counterpart (p<0.05). Similarly, more experienced pharmacists (> 10 years) held positive perceptions towards antimicrobial stewardship (p<0.05).

**Conclusion:**

The study highlighted some gaps in the perception and practices of community pharmacist towards antimicrobial stewardship. Development of customized interventions would be critical to bridging these gaps and improve their perception and practices towards antimicrobial stewardship.

## Introduction

The increasing prevalence of multi-drug resistant bacteria is leading to increasing mortality from otherwise treatable infections. It is estimated that around 26,000 people are dying from multi-drug resistant bacteria in the United States each year [1, 2]. Mortality in other parts of the world is also alarming with about 25,000 patients die each year in European Union countries from infections caused by multi-drug resistant bacteria [3]. Multi-drug resistance is estimated to result in about 96,000 deaths in Southern Asia [4]. A number of factors are responsible for the increasing antimicrobial resistance. Nevertheless, inappropriate use of antimicrobials is one of the modifiable factors. Methicillin-resistant *Staphylococcus aureus* (MRSA) is the most common marker for multidrug-resistance pathogens [5]. In Malaysia, the Ministry of Health report indicated that the rate of MRSA ranged from 0.4 to 35.8%, with an overall rate of 19.2% [6]. Several researchers have highlighted the association between the use of antibiotic and its resistance, both at the individual and community level [7]. Antimicrobial resistance significantly imposes the burden on the healthcare system as it is associated with higher morbidity and mortality rate, and prolonged length of hospital stay [8].

The term ‘Antimicrobial stewardship’ (AMS) is collectively used for a number of quality improvement activities focused on increasing and sustaining the appropriate use of antimicrobials for the treatment and/or prevention of infectious diseases. Given the high prevalence of multidrug-resistant bacteria globally, there has been an increasing interest in the implementation of AMS programs [9]. The majority of AMS programs have been introduced in institutional settings and comparatively, little attention has been paid to the replication of same initiatives in community practice. In Europe, the antibiotic use has been varied by a factor of 3.8 between the countries with the highest use in Greece (38.6 defined daily dose per 1000 inhabitants) and the lowest use in Romania (10.2 defined daily dose per 1000 inhabitants) [10]. In the United States (US), an estimated 1.4 billion outpatient antibiotics were dispensed from 2000 to 2010. Antibiotic prescription rates in the US remained relatively stable during this period from 382 per 1,000 persons in 2000 to 384 per 1,000 persons in 2010 [11]. As such, community pharmacists can play an important role in the development and implementation of AMS programs within community settings.

In Malaysia, antibiotics accounted for the largest proportion of money spent in 2006 and 2007 [12]. A local study revealed inadequate compliance of community pharmacists with the concept of rational use of drugs since 25% of community pharmacists dispensed antibiotics without prescriptions based on the presentation of infectious disease symptoms [13]. In view of emerging antimicrobial resistance, researchers have encouraged the healthcare professionals to hold the responsibility of AMS in practice settings in Malaysia [12]. Given the potential role of community pharmacists in the development and implementation of AMS program, it is important to understand the perceptions of community pharmacists towards AMS. The aim of this study was to assess pharmacists’ perception and practices towards AMS in community pharmacy settings in the State of Selangor, Malaysia.

## Ethical approval

The study was reviewed and approved by The Institutional Review Board of the Discipline of Social and Administrative Pharmacy, School of Pharmaceutical Sciences, University Sains Malaysia. Furthermore, written consent was taken from the participants prior to data collection. High level of confidentiality and anonymity was maintained throughout the study.

## Methods

### Study design, settings and sampling

A descriptive, cross-sectional study was conducted for the period of 2 months from March to April, 2015, in the State of Selangor. It is one of the most prosperous states in Malaysia, with a population of about 3.75 million inhabitants. A list of all the registered community pharmacies in Selangor was obtained from Malaysian pharmaceutical Society (MPS) (n = 297). Based on a pilot survey, there was an expectation of receiving 1.24 responses per pharmacy. A sample size was calculated through Raosoft software [14] in which the population was taken as 367; power was kept as 80%, response distribution as 50%, while confidence interval and margin of error was set at 95% and 5% respectively. A total of 188 pharmacists were needed to be sampled to generalize the findings. Pharmacy sites were randomly chosen for data collection using a simple random sampling approach based on the available list from MPS [15]. The process of data collection was continued until the required sample size was achieved. Data collectors visited the randomly selected community pharmacies; fully registered pharmacists were identified and were asked for their willingness to participate in this study. Provisionally registered pharmacists (trainee) were excluded from this study.

### Study Instrument

A self-administered questionnaire was designed after a thorough literature review of the relevant published studies [11–13, 16–18] (S1 Text). An initial version of the questionnaire was subjected to content and face validity. For content validity, the questionnaire was sent to a panel of 3 subject experts for their opinion on the relativity and the importance of the content. Necessary adjustments were made to the questionnaire prior to administering it to a small group of 10 community pharmacists for their suggestions on making the questionnaire more brief and simple. The proposed changes were integrated into the questionnaire while ensuring its consistency with the published literature. Reliability coefficient was calculated by using SPSS v.20 and the value of Cronbach’s alpha was calculated as 0.72 and 0.74 for perceptions and practices section respectively. The data of the pilot study was not used for the final analysis. The final instrument consisted of 24 items which were divided into three sections. The first section included 5 questions which explored the demographic information of the participants. The second section, comprised of 8 questions, assessed the perceptions of participants towards AMS. The responses of the participants in this section were recorded on 5 points Likert scale of agreement. A score of 1 was given to strongly disagree, 2 to disagree, 3 to Neutral, 4 to agree and 5 to strongly agree. The last section evaluated the practices of participants towards AMS by assessing their responses towards 11 questions. A 5 points Likert scale level of frequency was used to record participants’ practices towards AMS. A score of 1 was given to never, 2 to rarely, 3 to occasionally, 4 to often and 5 to always. Reverse coding was done for negatively worded statements.

### Data Analysis

Data were analysed by using SPSS v.20. Descriptive analyses were employed to express the data as frequencies and percentages. Kolmogorov-Smirnov and Shapiro-Wilks tests were carried out to test the normality of the data. Inferential statistics (Mann-Whitney and Kruskal-Wallis tests) were applied to detect the differences in median scores in view of the non-normal distribution of the data. Bonferroni adjustment was used to investigate the significance of intergroup variables. A p-value of less than 0.05 was considered as statistically significant.

## Results

A total of 225 pharmacists were approached to recruit a sample of 188 pharmacists, giving a response rate of 83.5%. The majority of participants were females (n = 103, 54.8%), and between the age group of 20–30 years (n = 144, 76.6%). The demographic profiles of the respondents are shown in Table 1.

**Table 1:** Participants’ characteristics.

Variable	Frequency	Percentage
Gender		
Female	103	54.8
Male	85	45.2
Age (in years)		
20–30	144	76.6
31–40	32	17
41–50	12	6.4
Qualification		
Bachelors	142	75.5
Masters	46	24.5
Experience (in years)		
<1	54	28.7
1–4	89	47.3
5–9	36	19.1
>10	9	4.8

The majority of pharmacists responded to the survey unanimously agreed (n = 182, 96.8%) that AMS program is essential to improve patient care (Median = 4; IQR = 1). A large proportion of participants agreed that pharmacists can play a prominent role in the development and delivery of antimicrobial stewardship programs (n = 175, 93.1%, Median = 5, IQR = 1). Moreover, respondents agreed or strongly agreed (n = 165, 87.7%) that conferences, workshops and other educational activities are important for the community pharmacists to enhance their understanding of AMS (Median = 4, IQR = 1). Contrary to the mostly positive views on the usefulness of AMS and the role of pharmacists, there was a visible lack of support for the incorporation of AMS programs at a community pharmacy level with more than half of the pharmacists reporting neutral views on this item (n = 102, 54.2%, Median = 3, IQR = 1). Similarly, the majority of participants (n = 59, 31.4%) were neutral in their opinion about the statement that individual’s efforts at antimicrobial stewardship have a minimal impact on antimicrobial resistance program (Median = 3, IQR = 2) (Table 2).

**Table 2:** Perception of participants towards antimicrobial stewardship.

Statements	Participants’ responses*					Median (IQR)
	SD	D	N	A	SA	
It improves patient care	0	0	6 (3.2)	94 (50)	88 (46.8)	4(1)
It should be incorporated at community pharmacy level	22 (11.7)	12 (6.3)	102 (54.2)	32 (17)	20 (10.6)	3(1)
It reduces problem of antimicrobial resistance	0	0	30 (16)	99 (52.7)	59 (31.4)	4(1)
Adequate training should be provided to community pharmacists on antimicrobial use	3 (1.6)	7 (3.7)	54 (28.7)	83 (44.1)	41 (21.8)	4(1)
Relevant conferences, workshops and other educational activity are required to be attended by community pharmacists to enhance understanding of antimicrobial stewardship	0	0	23 (12.2)	126 (67)	39 (20.7)	4(1)
Individual efforts at antimicrobial stewardship have minimal impact antimicrobial resistance problem	24 (12.8)	29 (15.4)	59 (31.4)	55 (29.3)	21 (11.2)	3(2)
I think that the prescribing physicians are the only professionals who need to understand antimicrobial stewardship	63 (33.5)	101 (53.7)	17 (9)	5 (2.7)	2 (1.1)	4(1)
pharmacists have a responsibility to take a prominent role in antimicrobial stewardship and infection prevent and control programs in the health system	0	0	13 (6.9)	68 (36.2)	107 (56.9)	5(1)

* SD = Strongly disagree, D = Disagree, U = Undecided, A = Agree, SA = Strongly agree. Note: Perception was assessed by giving 1 to SD, 2 to D, 3 to U, 4 to A, 5 to SA

A significant number of participants always (n = 73, 38.8%) or often (n = 95, 50.5%) communicate with prescribers in case of uncertainty about the appropriateness of antibiotic prescription (Median = 4, IQR = 1). Similarly, participants never (n = 69, 36.7%) or rarely (n = 89, 47.3%) dispense antimicrobial without a prescription; however, 19.1% (n = 36) pharmacists often, and 9% (n = 17) always dispense antibiotics for a duration longer than prescribed by the physicians on patient’s request (Median = 4, IQR = 1). Collaboration with other healthcare professionals on antimicrobial use is often done by pharmacists (n = 104, 55.3%) (Median = 4, IQR = 1). It was observed that 23.9% (n = 45) respondents rarely participate in antimicrobial awareness campaigns, while 30.3% (n = 57) pharmacists occasionally participate in such programs (Median = 3, IQR = 1) (Table 3).

**Table 3:** Practices of participants towards antimicrobial stewardship.

Statement	Participants’ responses					Median (IQR)
	Never	Rarely	Occasionally	Often	Always	
I dispense antimicrobial on prescription with complete clinical information	10 (5.3)	28 (14.9)	54 (28.7)	82 (43.6)	14 (7.4)	4 (1)
I dispense antimicrobials without a prescription.	69 (36.7)	89 (47.3)	10 (5.3)	18 (9.6)	2 (1.1)	4 (1)
I dispense antimicrobial for durations more than prescribed by the physician on patient’s request	24 (12.8)	74 (39.4)	37 (19.6)	36 (19.1)	17 (9)	4 (1)
I screen the antimicrobial prescription in accordance with local guidelines before dispensing	2 (1.1)	10 (5.3)	46 (24.5)	88 (46.8)	42 (22.3)	4 (1)
I collaborate with other health professionals for infection control and antimicrobial stewardship	5 (2.7)	9 (4.8)	54 (28.7)	104 (55.3)	16 (8.5)	4 (1)
I communicate with prescribers if I am unsure about the appropriateness of an antibiotic prescription.	0	2 (1.1)	18 (9.6)	95 (50.5)	73 (38.8)	4 (1)
I sought additional clinical information (e.g. drug interaction, ADRs, allergy, etc.) before deciding to dispense the antibiotic prescribed.	2 (1.1)	8 (4.3)	32 (17)	116 (61.7)	30 (16)	4 (1)
I take part in antimicrobial awareness campaigns to promote the optimal use of antimicrobials	9 (4.8)	45 (23.9)	57 (30.3)	67 (35.6)	10 (5.3)	3 (2)
I educate patients on the use of antimicrobials and resistance-related issues	6 (3.2)	12 (6.4)	36 (19.1)	115 (61.2)	19 (10.1)	4 (1)
I make efforts to prevent or reduce the transmission of infections within the community	18 (9.6)	2 (1.1)	64 (34)	86 (45.7)	18 (9.6)	4 (1)
I ask the patients about their knowledge of prescribed antimicrobial and its usage	4 (2.2)	10 (5.3)	36 (19.1)	106 (56.4)	32 (17)	4 (1)

Note: Practices was assessed by giving 1 to never, 2 to rarely, 3 to occasionally, 4 to often, 5 to always

Qualification of the participants significantly affected their median scores with respect to their perceptions (Median score: Bachelors = 3 vs Masters = 4) and practices (Median score: Bachelors = 3 vs Masters = 3.5) of AMS, while experience significantly affected the perceptions of participants towards AMS. Participants with less than one year of experience had significantly lower median scores than participants with 5–9 years of experience (Median scores: 3 vs 4, p<0.05), and participants with more than 10 years of experience (Median scores: 3 vs 4, p<0.05). Median scores of participants about their perceptions and practices of antimicrobial stewardship are tabularized in Table 4.

**Table 4:** Median scores of participants about their perception and experience of antimicrobial stewardship.

Variable	Perception score (Median)	Rank	P value	Practice score (Median)	Rank	P value
Gender*						
Female	4	95.7	0.691	4	92.9	0.62
Male	4	92.9		4	96.3	
Age (in years)**						
20–30	4	96.92		4	93.2	
31–40	4	77.38	0.144	4	94.6	0.514
41–50	4	91.08		4	109.5	
Qualification*						
Bachelors	3	92.8	0.013	3	78.5	0.036
Masters	4	143.75		3.5	95	
Experience (in years)**						
<1	3	73.6		4	83.2	
1–4	3.5	90.4	0.021	4	98	
5–9	4	104.7		4	100.9	
>10	4	109.2		4	101.2	0.227

* Mann Whitney Test

** Kruskal Wallis Test

## Discussion

The judicious use of antimicrobials is an essential approach to conserve the efficacy of infectious diseases. Pharmacists have a responsibility to take prominent roles in AMS and infection control programs in health systems. This study provides important insights into the perceptions and experiences of pharmacists towards AMS in community settings. The findings reinforce previous work indicating the expanding role of pharmacists in mediating antibiotic use internationally [19, 20]. The results of this study indicate positive perceptions and practices of community pharmacists towards AMS. Results of this survey identified qualification as a major determinant factor regarding the perceptions and practices of community pharmacists, while the experience of the participants significantly affected their perceptions towards AMS. To the best of our knowledge, the findings reported in this study give a first insight regarding the perceptions and practices of community pharmacists towards AMS in Malaysia.

In this study, community pharmacists recognized the importance of AMS as the majority were in agreement that AMS is essential to improve patient care. There is overwhelming evidence corroborating the notion that AMS is essential to optimize antimicrobial prescribing in order to improve patient care [21]. In Malaysia, Infection and Antibiotic Control Committees have been established at national, state and hospital levels. In 2014, a protocol on AMS program in hospitals and primary healthcare settings was developed by Ministry of Health Malaysia to optimize the use of antibiotics. Our findings are important amidst these crucial developments as the positive perceptions of pharmacists strengthen the concept of developing AMS programs for community pharmacies in Malaysia. The continuous monitoring of antibiotic dispensing by community pharmacists is an important strategy to contain antibiotic resistance. Since there is a limited literature, more studies are required to quantify a number of antibiotics dispensed by community pharmacists on a regular basis to optimize the use of antibiotics. The incentives behind inappropriate antibiotic dispensing need to be fully understood to design intervention strategies based on that knowledge. Further evidence supporting our results lie in the findings of Cotta et al. who reported that improving antimicrobial prescribing would reduce the incidence of resistance and improve patient care [18].

Pharmacists are greatly positioned to act as an effector arms for AMS programs because of their responsibility for processing medication orders [22]. The findings of the current study are in line with the other published studies [23] as a large proportion of participants agreed that pharmacist has a prominent role in AMS program in healthcare settings. However, it is noteworthy to mention that majority of respondents in this study were neutral in their opinion about the incorporation of AMS programs in community pharmacy settings. The likely reason for this discrepancy could be the under-utilization of community pharmacists in the war against antimicrobial resistance, although researchers indicate that community pharmacist could play an essential role in optimizing the use of antibiotics [24]. Furthermore, there is a growing support to incorporate AMS program at community pharmacy level [25]. Currently, there is no established AMS program that formally exists in community pharmacy settings in Malaysia. It is a positive indication that community pharmacists are aware of their roles in AMS programs; however, there is a need to utilize their positive understanding of AMS by including them in an inter-sectoral task force involving members from government, hospitals, the pharmaceutical industry, and the consumers. Stakeholders should provide recommendations for how community pharmacists can act as AMS proponents. Increase awareness of regional antibiotic resistance patterns, knowledge of latest treatment guidelines, limiting and adjusting the inventory of antibiotics are the important strategies that could be implemented as a part of community antimicrobial stewardship.

The findings of the current study highlight the challenge of making antimicrobial stewardship a relevant local issue among community pharmacists. Implementation of antimicrobial stewardship programs requires team efforts, and interdisciplinary involvement of different healthcare professionals. The participants of this study were neutral in their opinion of this point of view. In contrast, the findings of another study revealed that pharmacists were more in agreement to introduce a specialist multidisciplinary team giving advice on antimicrobial prescribing [18]. A shared decision making promotes the collaboration between pharmacists and other health professionals. Without this collaboration, pharmacists may not significantly influence the processes of improving antibiotic prescribing. Our findings indicate interprofessional issues that require attention if antibiotic stewardship roles are to be effective in community settings.

The findings suggest that a large proportion of pharmacists dispense antibiotics without a prescription. Financial incentives and business orientations of community pharmacies have been regarded as the major reasons of irrational dispensing of antibiotics [26, 27]. In Malaysia, high medicine prices are an important public health concern. Since the prices are not regulated, they vary considerably from one pharmacy to another [28]. More studies are required to investigate the effect of financial motives on the dispensing practices of community pharmacists. Researchers suggest that effective communication between pharmacist and physician is a core feature of antimicrobial stewardship [9]. The findings of current research validate this view as the majority of pharmacists communicate with prescriber in case of uncertainty about the appropriateness of antibiotic prescription [29]. Improved communication between healthcare professionals could significantly aid in optimizing the use of antibiotics. Several trials have established that longer duration of antibiotic therapy encourages the development of an antibiotic-resistant organism [30, 31]. However, more than quarter of participants revealed that they often or always dispense antibiotics for duration longer than prescribed by the physicians. Current research validates the findings of another Malaysian study reported elsewhere [13]. Efforts should be made to encourage pharmacists to adhere to the guidelines about the dispensing of antimicrobials. The results of present study show that majority of pharmacist rarely or occasionally participate in antimicrobial awareness campaigns. These findings are not in accordance with a study in which pharmacists were more willing to participate in AMS interventions in healthcare settings [18]. This highlights the need to encourage pharmacists to participate in antimicrobials awareness initiatives and enhance public and professional awareness on the importance of antimicrobial surveillance.

The overall practices of community pharmacists toward antimicrobial stewardship were positive. However, in Malaysia, the responsibility of medication dispensing is shared by general practitioners, who apart from their professional clinical duties also dispense medications [32]. The transformation of community pharmacy practice in Malaysia has been slow due to the nonexistence of a dispensing separation policy between pharmacists and medical doctors in private community practices. The most important reason for advocating dispensing separation is the higher risk of irrational prescribing by dispensing doctors. It has been reported that dispensing doctors prescribe 7 times more antibiotics than non-dispensing doctors in primary care clinics [33]. Dispensing separation is a critical policy change that would be essential to a successful antimicrobial stewardship program in Malaysia.

In this era of antimicrobial resistance, it is essential to incorporate AMS programs in community settings. Importantly, the effects of these AMS programs on antimicrobial resistance are still unknown in the greater part of the world [20]. Future large-scale studies that assess the effect of community AMS and clinically relevant outcomes, including antimicrobial resistance, are needed. The strength of this study is that it has highlighted an area where the availability of literature is limited. A state-wide study with the inclusion of participants through simple random sampling was also the strength of this study. However, cautions should be taken while interpreting these results in the context of potential identified limitations. The results may not be generalizable to all the community pharmacists in Malaysia as the sample was taken only from the state of Selangor. Since the participants were approached randomly to collect data, they may not account for the differences within the population. Although it does not reflect the internal validity of the findings, it may decrease the overall generalizability of the results. Despite the limitations identified, our findings have important implications for optimizing the use of antibiotics in community settings.

## Conclusion

Overall, the perceptions and practices of pharmacists toward antimicrobial stewardship were positive. However, there is a need for improvement in areas such as incorporation of antimicrobial stewardship program in community pharmacies, the importance of team work and interdisciplinary involvement of different healthcare professionals, and the participation of pharmacists in antimicrobial awareness campaigns. Further studies are warranted to validate these results by including pharmacists from other states of Malaysia. Interventions to improve the perception and practices of pharmacists should be customized to target weak areas highlighted in this study.

## Supporting Information

S1 Text Questionnaire used in the study. (DOCX)
