# Supplementary material for: Perceptions and Practices of Community Pharmacists towards Antimicrobial Stewardship in the State of Selangor, Malaysia
Source: PLoS One. 2016 Feb 22;11(2):e0149623. doi: 10.1371/journal.pone.0149623 (PMC4764324; doi:10.1371/journal.pone.0149623)
Supplement: S1 Text — (DOCX) [file pone.0149623.s001.docx]

**Perceptions and practices of community pharmacists towards antimicrobial stewardship in the State of Selangor, Malaysia**

Letters to participants,

We are currently conducting a pharmacoepidemiology study in relation to pharmacists' perceptions and practices towards antimicrobial stewardship in Malaysian community.

Antimicrobial stewardship refers to a coordinated intervention designed to enhance and improve the selection of an optimal antimicrobial drug regimen, dose, duration of therapy, and route of administration. It ultimately seeks to achieve an optimal therapeutic outcome in relation to the antimicrobial use, thus minimizing toxicity and other adverse events. This in turn reduces costs of health care for infections, thus limiting the occurrence of antimicrobial resistant strains.

Your participation is highly appreciated. The gathering of information will be kept confidential and used only for research purpose. The estimated completion time for this survey is 10 - 15minutes.

Anonymity and confidentiality: The data collected in the study will be treated as strictly confidential. The participants’ name would not be revealed and confidentiality of participants’ responses would be maintained by the researchers. Any of your information would not be release to your organization or anyone in a way that could identify you.

Right of withdrawal: Your participation in this research is voluntary. I do not anticipate any risks resulted from participating in this study other than minimal fatigue.

**Section A: Personal Information**

1. Please indicate your gender

|  | Male |
| --- | --- |
|  | Female |

1. Please indicate your age group

|  | 20-30 |
| --- | --- |
|  | 31-40 |
|  | 41-50 |
|  | 51-60 |
|  | Above 60 |

1. Please indicate your highest educational degree that you have attained at this point in time:

|  | Bachelor’s Degree in Pharmacy |
| --- | --- |
|  | Master’s Degree in Pharmacy |
|  | Doctorate Degree Pharmacy |
|  | Other, specify………. |

1. Please specify the number of years you have been practicing in this sector

|  | < 1 |
| --- | --- |
|  | 1-4 |
|  | 5-9 |
|  | ≥10 |

**Sector B: Perception of participants towards antimicrobial stewardship (AMS)**

| **Strongly Disagree – Strongly Agree (1-5)** |
| --- |

1. AMS programs improve patient care.

| **1** | **2** | **3** | **4** | **5** |
| --- | --- | --- | --- | --- |
| **Strongly Disagree** | **Disagree** | **Neutral** | **Agree** | **Strongly Agree** |
|  |  |  |  |  |

1. AMS should be incorporated at community pharmacy level

| **1** | **2** | **3** | **4** | **5** |
| --- | --- | --- | --- | --- |
| **Strongly Disagree** | **Disagree** | **Neutral** | **Agree** | **Strongly Agree** |
|  |  |  |  |  |

1. AMS programs reduce problem of antimicrobial resistance.

| **1** | **2** | **3** | **4** | **5** |
| --- | --- | --- | --- | --- |
| **Strongly Disagree** | **Disagree** | **Neutral** | **Agree** | **Strongly Agree** |
|  |  |  |  |  |

1. Adequate training should be provided to community pharmacists on antimicrobial use.

| **1** | **2** | **3** | **4** | **5** |
| --- | --- | --- | --- | --- |
| **Strongly Disagree** | **Disagree** | **Neutral** | **Agree** | **Strongly Agree** |
|  |  |  |  |  |

1. Relevant conferences, workshops and other educational activity are required to be attended by community pharmacist to enhance understanding of antimicrobial stewardship.

| **1** | **2** | **3** | **4** | **5** |
| --- | --- | --- | --- | --- |
| **Strongly Disagree** | **Disagree** | **Neutral** | **Agree** | **Strongly Agree** |
|  |  |  |  |  |

1. Individual efforts at antimicrobial stewardship has minimal impact antimicrobial resistance problem.

| **1** | **2** | **3** | **4** | **5** |
| --- | --- | --- | --- | --- |
| **Strongly Disagree** | **Disagree** | **Neutral** | **Agree** | **Strongly Agree** |
|  |  |  |  |  |

1. I think that the prescribing physicians are the only professionals who need to understand antimicrobial stewardship

| **1** | **2** | **3** | **4** | **5** |
| --- | --- | --- | --- | --- |
| **Strongly Disagree** | **Disagree** | **Neutral** | **Agree** | **Strongly Agree** |
|  |  |  |  |  |

1. Pharmacists have a responsibility to take prominent role in antimicrobial stewardship and infection control programs in health system.

| **1** | **2** | **3** | **4** | **5** |
| --- | --- | --- | --- | --- |
| **Strongly Disagree** | **Disagree** | **Neutral** | **Agree** | **Strongly Agree** |
|  |  |  |  |  |

**Sector B: Practices of participants towards antimicrobial stewardship**

| **Never – Always (1-5)** |
| --- |

1. I dispense antimicrobial on prescription with complete clinical information

| **1** | **2** | **3** | **4** | **5** |
| --- | --- | --- | --- | --- |
| **Never** | **Rarely** | **Occasionally** | **Often** | **Always** |
|  |  |  |  |  |

1. I dispense antimicrobials without a prescription..

| **1** | **2** | **3** | **4** | **5** |
| --- | --- | --- | --- | --- |
| **Never** | **Rarely** | **Occasionally** | **Often** | **Always** |
|  |  |  |  |  |

1. I dispense antimicrobial for durations more than prescribed by the physician on patient’s request

| **1** | **2** | **3** | **4** | **5** |
| --- | --- | --- | --- | --- |
| **Never** | **Rarely** | **Occasionally** | **Often** | **Always** |
|  |  |  |  |  |

1. I screen the antimicrobial prescription in accordance with local guidelines before dispensing.

| **1** | **2** | **3** | **4** | **5** |
| --- | --- | --- | --- | --- |
| **Never** | **Rarely** | **Occasionally** | **Often** | **Always** |
|  |  |  |  |  |

1. I collaborate with other health professionals for infection control and antimicrobial stewardship.

| **1** | **2** | **3** | **4** | **5** |
| --- | --- | --- | --- | --- |
| **Never** | **Rarely** | **Occasionally** | **Often** | **Always** |
|  |  |  |  |  |

1. I communicate with prescribers if I am unsure about the appropriateness of an antibiotic prescription.

| **1** | **2** | **3** | **4** | **5** |
| --- | --- | --- | --- | --- |
| **Never** | **Rarely** | **Occasionally** | **Often** | **Always** |
|  |  |  |  |  |

1. I sought additional clinical information (E.g. drug interaction, ADRs, allergy, etc.) before deciding to dispense the antibiotic prescribed.

| **1** | **2** | **3** | **4** | **5** |
| --- | --- | --- | --- | --- |
| **Never** | **Rarely** | **Occasionally** | **Often** | **Always** |
|  |  |  |  |  |

1. I take part in antimicrobial awareness campaigns to promote the optimal use of antimicrobials

| **1** | **2** | **3** | **4** | **5** |
| --- | --- | --- | --- | --- |
| **Never** | **Rarely** | **Occasionally** | **Often** | **Always** |
|  |  |  |  |  |

1. I educate patients on the use of antimicrobials, and resistance-related issues.

| **1** | **2** | **3** | **4** | **5** |
| --- | --- | --- | --- | --- |
| **Never** | **Rarely** | **Occasionally** | **Often** | **Always** |
|  |  |  |  |  |

1. I make efforts to prevent or reduce the transmission of infections within the community.

| **1** | **2** | **3** | **4** | **5** |
| --- | --- | --- | --- | --- |
| **Never** | **Rarely** | **Occasionally** | **Often** | **Always** |
|  |  |  |  |  |

1. I ask the patients about their knowledge of prescribed antimicrobial and its usage

| **1** | **2** | **3** | **4** | **5** |
| --- | --- | --- | --- | --- |
| **Never** | **Rarely** | **Occasionally** | **Often** | **Always** |
|  |  |  |  |  |
